# The Role of Plant Virus-like Particles in Advanced Drug Delivery and Vaccine Development: Structural Attributes and Application Potential

**DOI:** 10.3390/v17020148

**Published:** 2025-01-23

**Authors:** Esperanza Peralta-Cuevas, Igor Garcia-Atutxa, Alejandro Huerta-Saquero, Francisca Villanueva-Flores

**Affiliations:** 1 Centro de Investigación en Ciencia Aplicada y Tecnología Avanzada (CICATA), Unidad Morelos del Instituto Politécnico Nacional (IPN), Boulevard de la Tecnología No. 1036, Xochitepec 62790, Mexico; mperaltac2400@alumno.ipn.mx; 2 Computer Science Department, Universidad Católica de Murcia (UCAM), Av. de los Jerónimos, 135, 30107 Murcia, Spain; igarcia839@alu.ucam.edu; 3 Departamento de Bionanotecnología, Centro de Nanociencias y Nanotecnología, Universidad Nacional Autónoma de México (UNAM), Km. 107 Carretera Tijuana-Ensenada, Ensenada 22860, Mexico; saquero@ens.cnyn.unam.mx

**Keywords:** plant virus-like particles (pVLPs), cellular uptake, scalability, expression systems, surface modifiability

## Abstract

Plant virus-like particles (pVLPs) present distinct research advantages, including cost-effective production and scalability through plant-based systems, making them a promising yet underutilized alternative to traditional VLPs. Human exposure to plant viruses through diet for millions of years supports their biocompatibility and safety, making them suitable for biomedical applications. This review offers a practical guide to selecting pVLPs based on critical design factors. It begins by examining how pVLP size and shape influence cellular interactions, such as uptake, biodistribution, and clearance, key for effective drug delivery and vaccine development. We also explore how surface charge affects VLP–cell interactions, impacting binding and internalization, and discuss the benefits of surface modifications to enhance targeting and stability. Additional considerations include host range and biosafety, ensuring safe, effective pVLP applications in clinical and environmental contexts. The scalability of pVLP production across different expression systems is also reviewed, noting challenges and opportunities in large-scale manufacturing. Concluding with future perspectives, the review highlights the innovation potential of pVLPs in vaccine development, targeted therapies, and diagnostics, positioning them as valuable tools in biotechnology and medicine. This guide provides a foundation for selecting optimal pVLPs across diverse applications.

## 1. Introduction

The term “virus-like particle” (VLP) traditionally describes particles that resemble viruses structurally but lack infectious components. In fields like vaccine development and biotechnology, VLPs are engineered specifically to be non-infectious by omitting viral genomes, ensuring a high level of safety [1]. Plant virus-like particles (pVLPs) have recently emerged as critical tools in bionanotechnology. As non-infectious, virus-mimicking particles, pVLPs offer a safe and innovative platform for diverse applications. The progression of pVLP research from foundational structural studies to complex biotechnological applications underscores their critical role in addressing challenging issues, thanks to their self-assembling properties, inherent safety, and versatility for functionalization [2,3,4].

**Figure 1:**
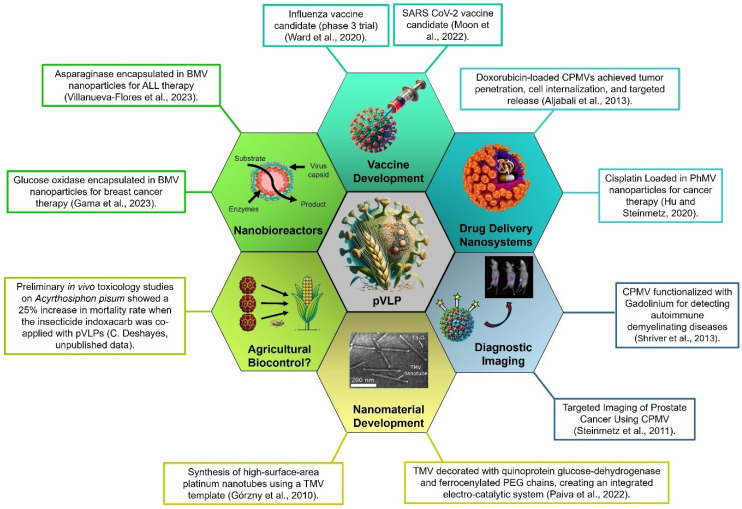
Summary of the principal biotechnological and biomedical applications of plant virus-like particles (pVLPs), showcasing key examples across diverse fields. These applications encompass vaccine development, targeted drug delivery, diagnostic imaging, nanomaterial engineering, nanobioreactors, and their potential role as synergistic agents in agricultural pest biocontrol. SARS-CoV-2: Severe Acute Respiratory Syndrome Coronavirus 2, CPMV: Cowpea Mosaic Virus, PhMV: Pepper Huanglongbing Mosaic Virus, TMV: Tobacco Mosaic Virus, PEG: Polyethylene Glycol, BMV: Brome Mosaic Virus, ALL: Acute Lymphoblastic Leukemia. References in the figure can be consulted [9,19,20,21,22,23,24,25,26,27].

Plant viruses’ structural flexibility and genetic diversity have enabled the development of pVLPs tailored to specific biotechnological needs. These particles offer distinct advantages over traditional therapeutic agents and vaccines, particularly in enhancing immune responses, which is crucial for vaccine development [5]. The ability of pVLPs to display multiple antigens, eliciting robust immune responses, has been particularly valuable in creating next-generation vaccines, demonstrating significant improvements in immunogenicity and vaccine efficacy [6,7]. Beyond their role in vaccinology, pVLPs have been explored as efficient vehicles for drug delivery, leveraging their capacity to encapsulate and deliver therapeutic molecules directly to target cells, thus minimizing off-target effects and enhancing therapeutic outcomes [8,9]. Figure 1 shows an overview of the most impactful biotechnological and biomedical applications of pVLPs, showcasing prominent examples in each domain. This summary underscores the broad utility of pVLPs across diverse fields, including vaccine development, targeted drug delivery, diagnostic imaging, nanomaterial engineering, bionanoreactor construction, development of innovative nanomaterials and their use as synergistic agents in agricultural pest biocontrol [6,10,11].

Numerous comprehensive reviews have recently been published, showcasing the broad range of applications for pVLPs [6,12,13,14,15,16,17,18]. Unlike these works, this review aims to provide a detailed guide for selecting the most suitable pVLPs based on these critical design aspects, ensuring optimal performance for specific applications. By examining the intricate relationship between pVLPs’ physicochemical properties and cell interaction, this review offers valuable insights for leveraging these particles to advance innovation and address pressing global challenges. Furthermore, it highlights future perspectives in the field, emphasizing the potential of pVLPs to drive significant advancements in nanomaterials for novel biotechnological and biomedical applications. Key factors in designing and applying pVLPs include their structural properties, size, shape, surface charge, surface functionalization, host range, biosafety, and production scalability. The structural properties of pVLPs, such as shape, size, and superficial charge, play a crucial role in determining their stability, cellular uptake, and overall functionality. Genetic engineering of pVLPs allows the display of specific antigens or the incorporation of therapeutic molecules, making them highly adaptable for various applications. Surface functionalization further enhances the versatility of pVLPs, enabling targeted delivery and diagnostic capabilities. Through a comprehensive understanding of these critical factors, researchers and industry professionals can make informed decisions that maximize the efficacy and applicability of pVLPs in diverse scientific and medical fields.

## 2. Key Considerations to Selecting Plant Viruses for Biotechnological or Biomedical Applications

When selecting plant viruses for biotechnological applications, it is essential to consider factors such as structural properties, including size, shape, surface charge, surface modifiability, host range, biosafety, and production scalability. Viruses like Tobacco Mosaic Virus (TMV) and Potato Virus X (PVX) are favored for their stable structures and ease of large-scale production, making them ideal for drug delivery and nanotechnology. Genetically modified and functionalizing the virus surface is essential for tailoring applications and ensuring biosafety, especially for human-related uses. Ultimately, the specific application will dictate the best choice, whether for vaccine development, therapeutic delivery, or creating nanostructures.

In the next section, we will explore the structural diversity of plant viruses and how their shape influences their properties, which is ultimately crucial in selecting the most suitable virus for specific biotechnological applications.

### 2.1. Impact of pVLPs’ Structural Attributes on Functionality and Applications

For biotechnological applications, building on the unique characteristics and safety profile of pVLPs, it is essential to consider how their structural attributes influence their functionality. Among these attributes, the shape of pVLPs plays a critical role in determining their physicochemical properties and biological interactions. The shape impacts vital factors such as cellular uptake, biodistribution, clearance from the body, tumor penetration, and overall drug delivery efficiency. Additionally, the immunogenicity of pVLPs can be significantly influenced by their structural form. In the following section, we will explore the influence of shape on the properties of pVLPs, providing a detailed comparison of different pVLP forms, including icosahedral, spherical, rod-shaped, and filamentous VLPs. Table 1 overviews various pVLP shapes, highlighting their structural and physicochemical characteristics, specific applications, and examples from existing literature. Understanding these aspects is critical for selecting the appropriate pVLP for biotechnological applications.

As illustrated in Table 1, each structure offers unique physicochemical properties and applications in biotechnology. For instance, icosahedral pVLPs are characterized by their symmetrical, spherical structure composed of 20 equilateral triangular faces, typically ranging from 20 to 200 nm in diameter. This symmetry confers high stability, making them suitable for accommodating larger proteins and epitopes. These properties make icosahedral pVLPs particularly valuable in vaccine development and drug delivery and as templates for synthesizing nanomaterials. Examples include the Cowpea Mosaic Virus (CPMV), Turnip Yellow Mosaic Virus (TYMV), and Red Clover Necrotic Mosaic Virus (RCNMV) [3,6,28].

Before addressing the differences between spherical and icosahedral viruses, it is helpful to define each one first. In virology, “spherical” and “icosahedral” describe related but distinct concepts. While most spherical viruses exhibit icosahedral symmetry, not all icosahedral viruses are strictly spherical. Icosahedral viruses comprise capsomers organized into an icosahedral structure, optimizing capsid stability through symmetric protein assembly [29]. In contrast, “spherical virus” refers to the general rounded appearance, which lacks strict icosahedral symmetry. This variation is seen in some flaviviruses, where symmetry shifts due to assembly factors and internal protein interactions [30].

The stability of icosahedral VLPs is attributed to their symmetrical structure, promoting uniform force distribution across the capsid and minimizing structural weaknesses. This geometry allows for strong non-covalent interactions between protein subunits, such as hydrogen bonds and hydrophobic interactions, enhancing stability under various environmental conditions. Indeed, icosahedral symmetry observed in virus capsids corresponds to potential energy minima that occur at specific “magic numbers” of particles, such as 12, 32, and 72 Lennard–Jones particles, where the packing achieves exactly twelve five-fold symmetry defects, an arrangement that optimizes stability and minimizes internal strain [31]. This configuration makes icosahedral VLPs particularly resilient to stressors such as pH shifts, temperature changes, and mechanical forces, making them ideal for biomedical applications that require high stability [32].

Spherical pVLPs often exhibit chemically reactive surfaces, allowing for functionalization and modification, which is advantageous for creating platforms to present target molecules for drug delivery. Some viruses, like Alternanthera mosaic virus (AltMV), which is a filamentous virus, however, can undergo thermal remodeling, transitioning from filamentous to spherical-like forms. This transition is confirmed through structural characterization techniques such as circular dichroism and fluorescence spectroscopy, revealing that the resulting spherical particles lack RNA and consist only of protein, distinguishing them from the original filamentous form [33]. Another example is the Tomato Spotted Wilt Virus (TSWV), which demonstrates the formation of spherical virus-like particles by expressing the TSWV glycoproteins Gn and Gc in plant cells. These proteins induce deformations in the endoplasmic reticulum and Golgi membranes, generating pleomorphic and spherical structures [34].

Spherical viruses have advantages in stability due to minimizing structural weaknesses and support even force distribution across its surface, thereby enhancing robustness under physical stress. This geometric arrangement also allows for stronger non-covalent interactions, such as hydrogen bonding, which adds resilience under conditions like changes in pH or temperature [35]. Furthermore, spherical shape contributes to energy efficiency during self-assembly, as it allows viral particles to achieve stable configurations that conserve genetic material while ensuring consistent shell formation [36]. This stability makes spherical viruses highly suitable for applications in biomedical research, where stability under diverse conditions is critical.

Otherwise, rod-shaped pVLPs exhibit elongated, cylindrical shapes with helical symmetry, varying in length from 100 nm to 2000 nm, with diameters typically around 10 to 20 nm. These particles are often rigid, providing mechanical robustness, and are beneficial in varied environmental conditions. Rod-shaped pVLPs encapsulate RNA genomes and are utilized in agricultural biocontrol, vaccine development, imaging technologies, and as models for studying virus assembly. Notable examples include TMV and PVX [28,37,38,39]. Their elongated structure, often with helical symmetry, provides increased stability under various environmental conditions, which withstand changes in pH and temperature without losing structural integrity. Additionally, rod-shaped viruses are highly suitable for functionalization in biomedical research, as their shape and multivalent surface allow for controlled tissue targeting and biodistribution, making them ideal for drug delivery and imaging applications [40]. Furthermore, the rigid capsid of some rod-shaped viruses provides a stable template for nanomaterial synthesis, where they serve as scaffolds for creating uniform nanostructures used in materials science and nanotechnology [41].

Finally, filamentous pVLPs are long, thread-like, and flexible, with helical symmetry. Their flexibility is advantageous in applications requiring dynamic structural changes, and they are suitable for high-density epitope presentation. These pVLPs are highly infectious and stable under various environmental conditions, making them ideal for vaccine development, studying viral replication, and creating nanowires and nanorods in nanotechnology. Examples include Flexuous viruses like Closteroviruses and TMV [28,42,43]. Filamentous viruses like certain bacteriophages are highly stable, even under stressors such as varying temperatures and pH levels, due to robust structural interactions among their coat proteins [44]. In addition, filamentous structures allow for enhanced interaction surfaces, improving binding and targeting for biomedical applications, such as in vaccines and nanomaterials [45].

In summary, the structural diversity of pVLPs offers distinct advantages for biotechnological applications. Icosahedral pVLPs provide high stability, ideal for vaccines and drug delivery; spherical forms enable effective surface functionalization; rod-shaped particles offer rigidity for robust drug delivery and nanomaterial synthesis; and filamentous pVLPs support high-density epitope presentation, enhancing vaccine development. Recognizing these unique characteristics is essential for selecting the optimal pVLP type for specific applications. Next, we will explore how the structural properties of pVLPs affect cellular uptake, biodistribution and clearance, drug delivery efficiency, and immunogenicity.

**Table 1:** Structural characteristics, applications, and examples of diverse pVLPs.

pVLP Shape	Structural Characteristics	Physicochemical Characteristics	Applications	Examples	References
Icosahedral	Symmetrical capsid. Composed of 20 equilateral triangular faces. Generally, ranges from 20 to 200 nm in diameter.	High stability due to symmetrical structure. It can accommodate larger proteins and epitopes.	Widely used in vaccine development due to their robust structure. Serve as carriers for drug delivery. Utilized as templates for the synthesis of nanomaterials.	Cowpea Mosaic Virus (CPMV). Turnip Yellow Mosaic Virus (TYMV). Red clover necrotic mosaic virus (RCNMV). Hibiscus chlorotic ringspot virus.	[3,6,28].
Spherical	Rounded shell shape that may lack precise symmetry. Spherical viruses are often stable and compact due to their assembled protein subunits.	The surface of spherical plant viruses can be chemically reactive, allowing for functionalization and modification.	Platforms for presenting target molecules. Platforms for drug delivery.	Brome Mosaic Virus (BMV). Tomato Spotted Wilt Virus (TSWV). Tomato Bushy Stunt Virus (TBSV).	[29,33,34].
Rod-shaped	Elongated, cylindrical shapes. Helical symmetry. Length varies significantly, typically from 100 nm to 2000 nm. Diameter around 10 to 20 nm. Often rigid.	Typically encapsulate RNA genomes. It can be mechanically robust and suitable for varied environmental conditions.	Used in agricultural biocontrol to infect plant pathogens. Utilized in the development of vaccines due to their stability and immunogenic properties. Serve as contrast agents in imaging technologies. Serve as models for studying virus assembly and infection mechanisms.	Tobacco Mosaic Virus (TMV). Potato Virus X (PVX). Alfalfa Mosaic Virus (AMV). Papaya mosaic virus (PapMV). Zucchini yellow mosaic virus.	[28,37,38,39].
Filamentous	Long, thread-like particles. Flexible and can vary greatly in length. This flexibility is advantageous for applications requiring dynamic structural changes. Helical symmetry.	It can be highly infectious. Suitable for high-density presentation of epitopes. These viruses can withstand various environmental stresses, making them stable across different conditions.	Used in vaccine development due to their immunogenic properties. Employed in studying viral replication and plant–virus interactions. Their self-assembly into regular structures makes them ideal for creating nanowires and nanorods in nanotechnology and materials science.	Flexuous viruses like Closterovirus. Turnip Mosaic Virus. Cardamom mosaic virus (CdMV).	[28,42,43].

#### 2.1.1. Influence of pVLP Shape and Size on Cellular Uptake and Tumor Penetration

The shape of pVLPs plays a significant role in determining their cellular uptake, which is crucial for their effectiveness in drug delivery and other biomedical applications. Research indicates that pVLPs with higher aspect ratios, such as rod-shaped or filamentous forms, exhibit more efficient cellular uptake than spherical particles. This efficiency is attributed to their ability to align parallel to the cell membrane, thereby reducing the energy barrier for membrane wrapping and endocytosis [46]. Additionally, the elongated shape increases surface contact with cell membranes, facilitating internalization through mechanisms like clathrin-mediated endocytosis and other endocytic routes [47,48]. Studies on TMV-based VLPs have demonstrated that rod-like particles are internalized more efficiently and utilize different internalization pathways than spherical particles, underscoring the critical role of shape in designing effective delivery systems [49].

Furthermore, pVLPs with spiky surfaces, which mimic the topology of certain viruses, have enhanced membrane penetration and uptake efficiency because the spikes can perturb the lipid bilayer, easing the entry of particles into the cell [50]. Studies using dissipative particle dynamics simulations show that these spikes reduce lipid density around the particle and minimize vertical deformation of the bilayer, lowering the penetration force required compared to smoother surfaces [50]. Furthermore, the interaction between spiky particles and the lipid bilayer induces transient lateral membrane strain, enhancing diffusion and stability for internalization [51].

The size of pVLPs also plays a critical role in determining their cellular uptake efficiency. Studies have shown that smaller nanoparticles, typically 20–50 nm in diameter, are more efficiently internalized by cells than larger particles [52]. Thermodynamic models suggest that nanoparticles of this size optimize internalization efficiency due to a balance between receptor binding energy and membrane deformation costs [53]. At this optimal size, the energy released from ligand–receptor interactions on the nanoparticle surface compensates for the energy required to deform the cell membrane for entry. Particles larger than 50 nm face increased energy barriers, making them harder to internalize, while smaller particles may not engage enough receptors to trigger efficient uptake [54]. For instance, research on CPMV particles of about 30 nm diameter demonstrated that these smaller VLPs had a higher cellular uptake rate than larger particles derived from TMV, which can reach sizes up to 300 nm [55]. The smaller size of CPMV facilitates easier penetration through the cell membrane, leading to more efficient internalization and subsequent intracellular trafficking.

Understanding these structural and functional implications is critical for selecting and optimizing pVLPs for targeted purposes. While smaller pVLPs may be taken up more efficiently, larger pVLPs (greater than 200 nm) can sometimes have advantages depending on the application. For example, larger particles might be retained longer in the bloodstream, enhancing their potential for drug delivery applications where prolonged circulation time is beneficial. However, their uptake is less efficient due to steric hindrance and reduced mobility through the extracellular matrix [52,56,57].

By understanding at a molecular level how the size and shape of pVLPs influence cellular uptake, it is possible to design better vehicles that enhance tumor penetration for innovative therapeutics design. For instance, particles within the 20–50 nm size range can be directly delivered to the lymph nodes, where they interact with B cells. In contrast, larger particles, ranging up to 500 nm, are more likely to be taken up by antigen-presenting cells (APCs) at the injection site and transported to the lymph nodes [58]. Additionally, research has shown that elongated, rod-shaped, and filamentous pVLPs, such as those derived from TMV and PVX, have superior tumor penetration abilities compared to spherical particles. This is mainly due to their high aspect ratios, which enable them to navigate through the dense extracellular matrix of tumors more efficiently. Studies have demonstrated that TMV-based nanoparticles exhibit higher diffusion rates within tumor tissues, allowing for more effective delivery of therapeutic agents deep into the tumor mass [59]. Similarly, PVX has been shown to exhibit enhanced tumor homing and tissue penetration, particularly in the core of tumors, compared to spherical nanoparticles like CPMV [60]. The elongated shape of these pVLPs also allows for better interaction with the tumor microenvironment, leading to improved biodistribution and retention within tumor tissues. This ability to penetrate deeper into tumors and deliver therapeutic payloads more effectively makes filamentous and rod-shaped pVLPs particularly advantageous for cancer treatment applications, as they can enhance the overall therapeutic outcomes [61].

These findings underscore the critical importance of both size and shape in designing pVLPs for targeted cancer therapies. By optimizing these parameters, it is possible to enhance the delivery and efficacy of therapeutic agents within tumor tissues, particularly in challenging environments such as the tumor core.

##### The Helfrich Energy Model as a Tool for Selecting Optimal pVLP Shape to Maximize Cell Internalization

The Helfrich energy model is a key theoretical framework used to describe the elasticity of lipid bilayers, particularly the energy associated with membrane bending [62]. This model is essential for understanding how nanoparticles of different shapes perturb the membrane and helps predict the energy required for the membrane to wrap around a nanoparticle or incorporate it through endocytosis. The bending energy depends on the membrane’s curvature, which is influenced by the shape of the interacting nanoparticle. Thus, a lower Helfrich energy is more favorable for particle internalization, as it indicates that less energy is required for the cell membrane to wrap around and internalize the particle. The model treats lipid bilayers as flexible, two-dimensional surfaces capable of bending and curving. The total bending energy of the membrane, $E_{Helfrich}$, is determined by Equation (1):

(1) $$E_{Helfrich}=\frac{k_{c}}{2}{\left(2H-C_{0}\right)}^{2}+\bar{k}K$$

where $k_{c}$ and $\bar{k}$ are bending rigidities, a property indicating the membrane’s resistance to bending that depends on the constituents of the lipid bilayer [63,64,65]; H is the mean curvature, describing the degree of membrane bending at each point; $C_{0}$ is the spontaneous curvature, reflecting the membrane’s preferred curvature and K is the Gaussian curvature, indicating the intrinsic curvature of the surface. Determining the parameters in the Helfrich energy model involves combining experimental techniques and theoretical calculations. Techniques like cryo-electron microscopy (cryo-EM) or Atomic Force Microscopy (AFM) can be used to image the membrane’s topology, and subsequent analysis provides the Gaussian curvature [66]. Similar to bending rigidity, molecular dynamics simulations and theoretical models can be used to estimate the Gaussian curvature modulus and curvature (M. Hu et al., 2012), and spontaneous curvature can often be inferred from the composition of the lipid bilayer, as specific lipids have a natural tendency to curve [67].

Membrane curvature plays a crucial role in these interactions. Positive mean curvature indicates outward bending (convex), while negative curvature indicates inward bending (concave). The actual curvature H compared to the spontaneous curvature $C_{0}$ dictates the accumulation of bending energy, affecting how the membrane interacts with nanoparticles or VLPs. When nanoparticles interact with a cell membrane, they induce local curvature changes based on shape and size. Spherical nanoparticles cause isotropic (uniform) bending, whereas elongated or rod-shaped nanoparticles create anisotropic (direction-dependent) bending [62].

This understanding is critical for designing nanoparticles and VLPs for drug delivery, as it enables researchers to optimize the shape and size of these particles for efficient cellular uptake.

#### 2.1.2. Influence of the Structural Properties of pVLPs in Biodistribution and Clearance

The shape of pVLPs plays a crucial role in determining their biodistribution and clearance in vivo, which are critical factors for their effectiveness in biomedical applications such as drug delivery. Studies have shown that elongated, rod-shaped, and filamentous pVLPs, such as those derived from the PVX and TMV, tend to have distinct biodistribution patterns compared to spherical particles. These rod-shaped pVLPs often show higher accumulation in specific organs like the liver and spleen and slower clearance rates, which can enhance their therapeutic potential by prolonging their presence in target tissues [68].

For example, a detailed analysis of PVX in mouse models revealed that these filamentous particles were primarily cleared by the reticuloendothelial system, particularly the spleen and liver, and exhibited slower clearance through the kidneys and bile, suggesting that the elongated shape of PVX allows for more effective tissue diffusion and retention than spherical particles, which are often cleared more rapidly from circulation [69].

Additionally, the interaction of these pVLPs with plasma proteins, known as the protein corona effect, can further influence their biodistribution and clearance. Rod-shaped pVLPs have been shown to interact differently with plasma proteins compared to spherical particles, affecting their dispersion, targeting, and clearance by the mononuclear phagocytic system [70].

However, the impact of pVLP size on biodistribution and clearance still needs to be explored. Some studies have shown that smaller nanoparticles (around 20–30 nm) tend to circulate longer in the bloodstream, showing delayed clearance and increased tumor accumulation due to enhanced permeation and retention effects, as opposed to larger particles, which are more readily captured by the liver and spleen, leading to faster clearance [71]. However, pharmacodynamic studies involving pVLPs are particularly scarce. This lack of data represents a significant gap in our understanding and limits the optimization of pVLPs for drug delivery and other therapeutic applications. Further research is needed to clarify how the size of pVLPs influences their biodistribution and clearance, which will be crucial for developing more effective nanocarriers tailored to specific biomedical needs.

#### 2.1.3. Impact of pVLP Size and Shape on Drug Delivery Efficiency

The shape of pVLPs plays a significant role in determining their drug delivery efficiency, particularly in how they interact with biological systems and transport drugs to target sites. Rod-shaped and filamentous pVLPs, such as those derived from the TMV and PVX, have shown distinct advantages in drug delivery applications due to their elongated structures, which facilitate better interaction with cell membranes and deeper penetration into tissues, including tumors. For instance, the elongated shape of TMV-based nanoparticles allows for more efficient delivery of chemotherapeutic agents to cancer cells, enhancing therapeutic outcomes by improving cellular uptake and intracellular trafficking [72].

Otherwise, spherical pVLPs, often having higher drug-loading capacities due to their structure, may not perform as well in navigating dense tissue environments like tumors. However, their ease of modification and ability to encapsulate various types of drugs make them valuable in other contexts, such as systemic delivery, where rapid distribution is necessary [73]. In addition to shape, the size of pVLPs is equally critical in determining drug delivery efficiency. However, this area is less explored in pVLPs than in their mammalian counterparts. Research on mammalian virus-derived VLPs has shown that smaller particles (typically under 100 nm) are more likely to penetrate deeper into tissues, including tumors, due to their ability to navigate the interstitial spaces more effectively [74,75]. This characteristic could be advantageous for delivering drugs to the hypoxic cores of tumors, where larger particles might be excluded. Conversely, larger VLPs (over 200 nm) tend to remain in circulation longer, which can benefit applications requiring sustained drug release. However, they may face challenges in penetrating dense tissues and avoiding rapid clearance by the reticuloendothelial system [76].

Unfortunately, detailed studies on the impact of pVLP size on drug delivery efficiency are lacking, representing a significant gap in the current literature. While the principles derived from mammalian VLPs provide some insights, it is crucial to investigate how these findings translate to pVLPs, given their different structural and biological characteristics.

#### 2.1.4. Impact of pVLP Shape and Size on Immunogenicity

VLPs are inherently immunogenic due to their structural similarity to actual viruses. This resemblance triggers the immune system to recognize and respond to VLPs as pathogens, eliciting strong immune responses. The repetitive and highly ordered surface structure of VLPs facilitates crosslinking of B cell receptors, leading to potent B cell activation and antibody production. Moreover, VLPs are readily taken up by APCs, which process and present VLP-derived antigens to T cells, further enhancing the immune response [77,78]. The shape of VLPs significantly impacts their immunogenicity, influencing how effectively they can stimulate an immune response. Studies have shown that the geometric shape of VLPs can alter their interaction with the immune system, particularly in how APCs process and present them.

For example, icosahedral pVLPs exhibit enhanced drainage to secondary lymphoid organs. This structure facilitates efficient activation of B cells and other immune components, often resulting in a more robust systemic immune response, including elevated levels of IgG and IgA antibodies, compared to rod-shaped VLPs [79].

Conversely, filamentous and rod-shaped VLPs, like those derived from the TMV and PVX, have been noted for their potential to induce strong immune responses due to their larger surface area, which provides more binding sites for immune cells. This shape allows for a high-density presentation of epitopes, which can enhance the immunogenicity of the VLPs, making them potent candidates for vaccine development [80]. These findings highlight the importance of VLP shape in the design of vaccines and immunotherapies, as different shapes can offer unique advantages depending on the desired immune response and application.

The size of pVLPs is also a critical factor in determining their immunogenicity. In virus-derived VLPs from mammals, smaller particles are more likely to induce Th2-type responses associated with humoral immunity. Conversely, larger particles typically elicit Th1-type responses, vital for activating cellular immunity, and play a key role in targeting and eliminating intracellular pathogens and cancer cells [81,82]. However, whether these observations hold for pVLPs remains unclear, highlighting the need for further research.

Having discussed the physical properties of pVLPs, such as size and shape, and their impact on biomedical applications, we will now turn our attention to other critical factors: the influence of surface charge on VLP–cell interactions, surface modifiability, host range and biosafety, and scalability in different expression systems. These factors are essential for tailoring pVLPs to specific biotechnological purposes, ensuring their effectiveness and safety in diverse applications. Understanding and optimizing these aspects are critical to successfully deploying pVLPs in various fields.

### 2.2. Impact of Surface Charge on VLP–Cell Interactions

The surface charge of a VLP significantly influences its interaction with cells, affecting its internalization efficiency and biodistribution. This surface charge can alter how VLPs behave in biological environments by modifying interactions with cell membranes, protein adsorption (protein corona formation), and recognition by specific cell surface receptors [70].

VLPs with a positive surface charge tend to interact more strongly with cell membranes, which are generally negatively charged due to the presence of phospholipids in the lipid bilayer. This electrostatic attraction facilitates the adsorption of VLPs to the cell surface, promoting their internalization through clathrin-mediated or caveolin-mediated endocytosis. In contrast, VLPs with a negative charge may experience electrostatic repulsion, potentially reducing their internalization efficiency. Studies have shown that nanoparticles with a positive charge exhibit higher cellular uptake rates than those with negative or neutral charges [52,56].

The Derjaguin–Landau–Verwey–Overbeek (DLVO) theory and Poisson–Boltzmann equation are critical tools for understanding how the external surface charge of VLPs influences their internalization into cells (Equation (2)). The DLVO theory combines two primary forces: van der Waals forces (which are attractive) and electrostatic forces (which can be either attractive or repulsive depending on the charges involved). Van der Waals forces are weak, non-specific forces that cause attraction between particles at very close distances. They can promote the initial contact between the VLP and the cell membrane. If the VLP and the cell membrane have opposite charges, electrostatic attraction will occur, making it easier for the VLP to adhere to the membrane. If they have like charges, electrostatic repulsion will create an energy barrier the VLP must overcome to get close enough to the membrane for internalization [83,84].

(2) $$\beta U\left(r\right)=Z^{2}\lambda_{B}{\left(\frac{e^{ka}}{1+ka}\right)}^{2}\frac{e^{ka}}{r}$$

where $\lambda_{B}$ is the Bjerrum length; U is the potential energy; e ≈ 2711828 is the Euler number; k is the inverse of the Debye–Hückel screening length ($\lambda_{D}$); k is given by $k^{2}=4\pi \lambda_{B}n$ and $\beta^{-1}=k_{B}T$ is the thermal energy scale at absolute temperature T.

Otherwise, the Poisson–Boltzmann equation helps model how the electric potential is distributed around a charged particle, such as a VLP, in a biological fluid. This equation considers the VLP’s surface charge and the surrounding ionic environment (e.g., the cell culture medium) (Equation (3)).

(3) $$\nabla^{2}\psi =\frac{c_{0}e}{\epsilon \epsilon_{r}}\left(e^{\frac{e\psi (x,y,z)}{k_{B}T}}-e^{-\frac{e\psi (x,y,z)}{k_{B}T}}\right)$$

where $\nabla^{2}\psi$ represents the spatial variation of the electric potential *ψ*; the electric work required to bring a charged cation to a place with potential *ψ* is W^+^ = eψ, and for an anion, it is W^−^ = −eψ; ψ(x,y,z) is the potential distribution that depends on the position; the local anion and cation concentrations c^−^ and c^+^ are related with the local potential *ψ* by the Boltzmann factor: $c^{-}=c_{0}e^{e\psi /k_{B}T}$ and $c^{+}=c_{0}e^{-e\psi /k_{B}T}$, where $c_{0}$ refers to the bulk concentration of the salt in the solution; ϵ and $\epsilon_{r}$ are the total relative permittivity and the dielectric permittivity in a medium [85]. Equation (3) calculates the electric potential field around the VLP. This field influences how the VLP interacts with the cell membrane, which has its charge distribution and helps predict the potential energy barrier that must be overcome for the VLP to approach and interact with the cell membrane. If the surface charge of the VLP is optimized (e.g., slightly positive to match the slightly negative charge of most cell membranes), the energy barrier for internalization is reduced, making the process more efficient.

The surface charge also affects the protein corona formation, a layer of proteins that adheres to the surface of VLPs when they interact with biological fluids such as blood. The composition of this corona can change depending on the surface charge of the VLP, which can alter its recognition by immune cells and its biodistribution in the body. For example, positively charged nanoparticles tend to attract specific proteins that may favor opsonization and subsequent phagocytosis by macrophages, potentially limiting their efficacy in therapeutic applications requiring prolonged bloodstream circulation [86].

Surface charge also influences the recognition of VLPs by cell receptors. For instance, positively charged VLPs can more easily interact with negatively charged receptors, such as heparan sulfate proteoglycans, which are widely distributed on the surface of various cells and mediate the entry of viral particles and nanoparticles [87].

The interaction between cell membranes and VLPs can be exploited to target VLPs to specific cell types, enhancing the selectivity and efficacy of VLP-based therapies. However, most of the current understanding of these interactions comes from studies on VLPs derived from mammalian viruses. While studies explicitly examining this in the context of pVLPs are sparse, the existing knowledge can likely be extrapolated to pVLPs, suggesting similar potential for targeted delivery and therapeutic applications. Nonetheless, more targeted research is needed to confirm these assumptions and optimize pVLPs for specific biomedical uses.

### 2.3. Capability of Genetic Modification and Surface Functionalization

Genetic modification of pVLPs involves altering the viral genome to include foreign genetic sequences that encode functional peptides, proteins, or other bioactive molecules. This process is critical for enhancing the specificity and efficacy of pVLPs in biomedical applications. Both genetic modifiability and surface functionalization are vital factors that significantly enhance the versatility and application potential of pVLPs in biotechnology. By engineering these particles at the genetic level and applying sophisticated surface modifications, researchers can tailor pVLPs for specific purposes, such as targeted drug delivery, vaccine development, and diagnostic imaging. This section underscores the inspiring role of genetic modification and surface functionalization in enhancing the versatility and application potential of pVLPs, motivating the audience to explore their potential further.

TMV is one of the most extensively studied plant viruses for genetic modification, primarily due to its well-defined structure and ease of manipulation. It has also been widely used because of its robust ability to incorporate foreign peptides into its coat protein. For instance, TMV’s coat protein can be genetically engineered to display peptides or entire proteins, creating chimeric particles that maintain their structural integrity while presenting new functionalities [88].

A study by Plummer et al. (2012) explored the limits of genetic modification in TMV, finding that while smaller peptides could be successfully incorporated without affecting the stability of the capsid, larger proteins often resulted in misfolding or aggregation of the particles. To mitigate this, researchers have developed strategies such as linker sequences or flexible regions within the capsid protein to accommodate larger inserts [89].

Otherwise, Koudelka et al. (2015) successfully inserted a peptide derived from the foot-and-mouth disease virus into the TMV coat protein, creating a chimeric VLP that retained the original structural integrity of the TMV while displaying the foreign antigen. The modified VLP elicited a robust immune response in animal models, underscoring its potential as a vaccine candidate. Additionally, TMV was modified to display a peptide from the human epidermal growth factor receptor 2 (HER2) on its surface, aiming to create a targeted delivery system for breast cancer therapy. The chimeric TMV particles could specifically bind to HER2-positive cancer cells, demonstrating the feasibility of using genetically modified pVLPs in targeted cancer therapy [90,91].

Koch et al. (2016) explored the stability of TMV-based virus-like particles (VLPs) following extensive genetic modification. They found that while the helical arrangement of TMV’s coat proteins provided a robust framework, certain genetic modifications could destabilize the capsid. To counteract this destabilization, the researchers introduced stabilizing mutations into the TMV coat protein, successfully preserving the structural integrity of the VLPs, even after significant genetic alterations [92].

Noad and Roy (2003) have discussed the broader implications of VLP stability in vaccine development. They noted that unstable VLPs might fail to elicit a robust immune response due to premature disassembly or degradation before reaching their target in the body. VLPs’ instability underscores the importance of structural integrity throughout the production, storage, and delivery processes [93].

In addition to genetic stabilization strategies, advancements in chemical crosslinking and encapsulation techniques have been employed to enhance VLP stability. For example, research by Peabody et al. (2008) demonstrated that chemical crosslinking of capsid proteins could significantly improve the thermal and mechanical stability of VLPs, making them more resilient to environmental stresses encountered during storage and administration [94].

To overcome the stability challenges associated with genetic modification and the inherent limitations of the expression system, surface chemical functionalization provides an efficient and selective method for enhancing the surface properties of pVLPs, allowing for enhanced functionality while maintaining capsid stability. One of the most used methods for surface functionalization is click chemistry, particularly the Cu(I)-catalyzed azide-alkyne cycloaddition (CuAAC). This reaction is favored due to its high efficiency, selectivity, and mild conditions, making it ideal for bioconjugation. Rubino et al. (2012) utilized CuAAC to attach fluorescent dyes and targeting ligands to TMV-based VLPs. This modification enabled the particles to bind specifically to cancer cells and visualize them using fluorescence microscopy, demonstrating the potential of surface-functionalized pVLPs in diagnostic imaging [95].

Advanced surface functionalization techniques have further expanded the capabilities of pVLPs, allowing for the creation of multifunctional platforms capable of carrying multiple payloads simultaneously. For example, Cho et al. (2014) developed CPMV-based nanoparticles that were functionalized with both therapeutic peptides and imaging agents, resulting in a dual-purpose platform for the simultaneous treatment and monitoring of cancer. The study found that these multifunctional pVLPs exhibited enhanced targeting and therapeutic efficacy compared to single-function particles [96].

In conclusion, integrating genetic modification with advanced surface chemical functionalization techniques provides a powerful approach to enhancing the functionality and stability of pVLPs. By addressing the limitations of genetic modifiability and employing methods like click chemistry, researchers can create multifunctional pVLP platforms that retain structural integrity while being tailored for specific biomedical applications. These advancements improve the targeting and efficacy of pVLPs and open new avenues for their use in complex therapeutic and diagnostic settings, demonstrating their potential as versatile tools in biotechnology.

### 2.4. Host Range and Biosafety

The selection of plant viruses for biotechnological applications requires careful consideration of host range and biosafety to ensure the safety and efficacy of these technologies while minimizing potential environmental impacts and unintended cross-species transmission. For example, Cucumber mosaic virus (CMV) is known to infect over 1200 species across more than 100 plant families, making it a significant example of cross-species transmission. Aphids commonly spread this virus in a non-persistent manner, where the insect quickly transfers the virus from an infected plant to a healthy one. A documented case involved the transmission of CMV from *Nicotiana tabacum* to *Capsicum annuum* via aphid vectors, resulting in significant crop losses in areas where both plants were grown together. CMV’s broad host range and adaptability pose a considerable threat to agriculture, making it essential to consider cross-transmission risks when selecting plant viruses for biotechnological or agricultural applications. These risks are particularly relevant in environments where multiple plant species coexist, as unintended host jumps could have severe consequences for non-target crops and ecosystems. Understanding the evolutionary dynamics of host range is crucial for selecting pVLPs that minimize these risks and ensure safe and effective use in diverse settings [97,98].

Phylogenetic studies provide insights into the evolutionary history of host range expansions and contractions. McLeish et al. (2019) discussed how intrinsic factors, such as genetic traits determining virus fitness, and extrinsic ecological factors, like species distribution and abundance, influence host range evolution in plant viruses [99]. Such analyses are invaluable for selecting pVLPs with a narrow host range, reducing the likelihood of affecting non-target species. Moreover, Moury and Desbiez (2020) emphasized that frequent host range changes during potyvirus evolution suggest that even closely related virus strains can exhibit significant differences in host specificity, underscoring the need for thorough host range assessments when developing pVLP-based applications [100].

Biosafety is a paramount consideration in developing pVLPs, mainly when these particles are intended for use in environments where they could interact with a range of organisms. Fraile et al. (2014) explored the impact of resistance-breaking mutations in tobamoviruses on virus survival and environmental stability. Their findings suggested that mutations conferring the ability to infect resistant host genotypes could also influence virus stability and persistence in the environment [101].

The ecological impact of pVLPs must be carefully evaluated, mainly when they are released into the environment as part of agricultural or pest control strategies. As researchers, scientists, and professionals in virology, genetics, and ecology, the role in this evaluation is crucial. Barratt et al. (2016) developed the Prioritization of Non-Target Invertebrates (PRONTI) model to prioritize non-target species for host range testing through a risk-based evaluation process, considering factors such as phylogenetic proximity and habitat ecology. Species with the highest risk of being affected by a biological control agent are selected for experimental testing, ensuring that ecological impact is minimized [102].

Regulatory frameworks for pVLP use in biotechnological applications require comprehensive risk assessments considering both host range and biosafety. The development of standardized protocols for testing pVLPs in diverse environments is essential for ensuring these particles can be safely deployed. McLeish et al. (2018) suggested that future research should focus on the interactions between viruses and their hosts in complex ecosystems, considering biotic and abiotic factors that influence host range evolution and disease emergence [99]. By integrating phylogenetic analysis, ecological impact assessments, and regulatory frameworks, researchers can develop pVLPs that are both effective in their intended applications and safe for the environment and non-target species. This approach will enable the responsible deployment of pVLPs in various biotechnological contexts, from agriculture to medicine.

### 2.5. Scalability in Different Expression Systems

The scalability of pVLPs is critical in their development and application in biotechnology, particularly for large-scale production. The choice of expression system is pivotal, as it influences not only the yield and cost but also the ability to perform post-translational modifications, which are essential for the functionalization of pVLPs. This section explores the scalability of pVLP production across various expression systems, including *Escherichia coli*, yeast, insect cells, plant systems, and mammalian cells, with a focus on the advantages and limitations of each.

#### 2.5.1. *Escherichia coli*

*Escherichia coli* is a widely used expression system due to its rapid growth, ease of genetic manipulation, and low production costs. It is often employed to produce pVLPs, mainly when high yield and scalability are the primary goals. However, one significant limitation of *E. coli* is its inability to perform post-translational modifications, such as glycosylation, which are often crucial for the functionality of VLPs in therapeutic applications. Additionally, endotoxin contamination is a significant concern when using *E. coli* as an expression system. Endotoxins, components of the outer membrane of Gram-negative bacteria like *E. coli*, can induce inflammatory solid responses if not adequately removed, potentially leading to adverse effects in therapeutic applications, including fever, sepsis, or anaphylactic reactions [103,104]. Furthermore, proteins expressed in *E. coli* may risk allergic reactions due to improper folding or lack of necessary glycosylation patterns typical in mammalian systems [105]. Despite these challenges, *E. coli* remains a viable option for producing simpler pVLPs or when modifications are not required.

#### 2.5.2. Yeast (*Pichia pastoris*)

Yeast systems, particularly *Pichia pastoris*, offer a middle ground between bacterial systems’ simplicity and higher eukaryotic systems’ complexity. *Pichia pastoris* is advantageous for its ability to perform post-translational modifications, including glycosylation. Yeast systems are also highly scalable and cost-effective, making them suitable for producing more complex pVLPs. For example, Uhde-Holzem et al. (2015) reported the successful production of PVX-based VLPs in *Pichia pastoris*, with modifications that allow for displaying complex foreign proteins, achieving high yields while maintaining structural integrity. One of the main disadvantages of using this system is the limited ability to perform human-like post-translational modifications. This limitation can lead to proper protein folding and assembly challenges, potentially impacting the final product’s functionality. Additionally, this system is generally more suited for producing simpler VLPs, particularly those related to cell wall structures [106].

#### 2.5.3. Insect Cells (Baculovirus Expression System)

Insect cells, using the baculovirus expression vector system (BEVS), are another popular choice for pVLP production, especially when human-like post-translational modifications are required. This system allows for producing highly complex VLPs, including those with extensive glycosylation and folding patterns similar to mammalian cells. BEVS has been extensively used to produce various VLPs, including those based on the PVX and other plant viruses. The scalability of this system is well documented, with industrial-scale production possible. Uhde-Holzem et al. (2015) demonstrated the capability of this system to produce PVX-based VLPs with high yields, suitable for large-scale applications. One of the main disadvantages of this system is that it offers simpler N-glycosylation than mammalian cells, which can affect the final product’s functionality. Additionally, the system produces lower yields, comes with high production costs, and presents difficulties in scaling up the process. Moreover, there is also a risk of baculovirus contamination, which can compromise the quality and safety of the production [106].

#### 2.5.4. Plant-Based Systems (*Nicotiana benthamiana*)

Plant-based systems, particularly *Nicotiana benthamiana*, have emerged as a promising platform for pVLP production due to their scalability and cost-effectiveness. These systems are particularly advantageous for producing glycosylated proteins, often necessary to functionalize pVLPs. Plant systems can be rapidly scaled up, and the transient expression allows for the quick production of large quantities of VLPs. For example, Mallajosyula et al. (2014) demonstrated the successful production of TMV-based VLPs in *N. benthamiana*, which were modified to display the influenza hemagglutinin protein, achieving high levels of chimeric VLPs [107]. Ward et al. (2020) conducted the first large-scale study of a plant-derived human vaccine to assess the efficacy, immunogenicity, and safety of a quadrivalent influenza pVLP vaccine in adults aged 18–64 and older adults aged 65 and above. The results demonstrated that the vaccine was well tolerated, with no significant safety concerns identified in participants across both studies [27].

One of the main disadvantages of using chloroplasts is the lack of post-translational modifications, which can limit the functionality of the produced proteins. The process also involves the time-consuming task of generating stable transgenic plants, further complicating production. Additionally, the expression levels achieved are often low, leading to insufficient VLP assembly and stability. The system is generally suited for producing simpler VLPs, and various technical challenges associated with using transgenic plants must be addressed [106].

#### 2.5.5. Mammalian Cells

The production of plant virus-like particles (pVLPs) in mammalian cell systems offers several advantages, including the ability to perform complex post-translational modifications critical for correctly assembling and folding proteins. This system also allows the production of more complex VLPs, and the possibility of culturing cells in suspension can facilitate the scaling up of processes. However, there are significant disadvantages associated with this method. Mammalian cells generally have a low growth rate and require a long expression time, leading to low overall yields. Additionally, the production costs are typically high, and scaling up the process can be challenging. Another critical concern is the risk of contamination by mammalian pathogens, which further poses safety risks and complicates the production process [108,109]. As far as we know, pVLPs have not yet been produced in mammalian cells.

Overall, the most suitable expression system depends on the specific requirements of the pVLP application. For large-scale, cost-effective production of simpler pVLPs, *E. coli* is a strong candidate. *P. pastoris* balances applications that need moderate complexity and some post-translational modifications. Despite their higher costs, insect cells are the preferred option for producing highly complex, human-like VLPs. Plant-based systems, like *N. benthamiana*, offer significant advantages in scalability and cost-effectiveness, especially for producing glycosylated proteins. However, they may not be suitable for applications requiring precise human-like modifications. Therefore, no single system can be deemed universally better or worse; instead, the choice should be guided by the specific needs of the biotechnological application in question.

## 3. Future Perspectives of pVLPs in Biotechnology

As research on pVLPs advances, the field holds significant potential for transformative breakthroughs in biotechnology. While much of the current focus has been on VLPs derived from mammalian viruses, the unique potential of pVLPs has yet to be explored. However, there is growing recognition of their versatility and utility. Future research and development in this area will likely be shaped by critical factors such as optimizing design features, enhancing production scalability, and exploring new application domains.

One critical area for future development is optimizing pVLP structural properties and surface modifiability. As our understanding of the relationship between pVLP structure and function deepens, opportunities will arise to engineer particles with highly specific properties tailored to particular applications. The integration of computational modeling, synthetic biology, and advanced genetic engineering techniques could lead to pVLPs with enhanced stability, improved immunogenicity, and the ability to carry multiple therapeutic payloads simultaneously [4]. Developing novel surface functionalization techniques, such as bio-orthogonal chemistries and modular assembly strategies, will allow for more precise control over pVLP functionalization. These advancements are particularly promising for expanding the use of pVLPs in precision medicine and personalized therapies, where highly tailored therapeutic agents are required. Customizing pVLPs at the molecular level will also improve their effectiveness in targeted drug delivery and vaccine development, as well as in the development of platforms for diagnostic imaging [110].

Scalability remains critical for spreading pVLPs, particularly in commercial applications such as vaccine manufacturing and therapeutic interventions. Future research is expected to optimize existing expression systems and develop new platforms that combine high yield with cost-effectiveness and the capability to perform complex post-translational modifications. While plant-based systems like *N. benthamiana* offer promising scalability, further advancements are needed to address limitations related to human-like glycosylation and other post-translational modifications [111,112]. The future of pVLP production will likely see the development of robust manufacturing platforms capable of delivering vaccines and therapeutic agents to resource-poor areas with reduced production times and costs [12].

Beyond their established roles in vaccine development and drug delivery, pVLPs are poised to enter new application domains as our understanding of their capabilities expands. One up-and-coming area is using pVLPs to develop biosensors and diagnostic tools. Their ability to be functionalized with a wide range of ligands and their inherent biocompatibility make them ideal candidates for detecting specific biomolecules or pathogens with high sensitivity and specificity [113]. Moreover, pVLPs have also been proposed as innovative biomaterials for pest control thanks to their ability to enhance the effectiveness of pesticides through synergistic interactions [106,114]. Additionally, pVLPs could play a significant role in the emerging field of nanomedicine, where they could be used to develop targeted delivery systems for gene therapy or as carriers for novel therapeutic agents such as RNA-based drugs. Their potential for customization and scalability makes them well suited for these cutting-edge applications, where precision and effectiveness are crucial [4,8,115,116]. Their potential for customization makes pVLPs suitable for developing vaccines against a wide range of pathogens, including those that are difficult to target with traditional vaccine platforms. In oncology, pVLPs could be instrumental in advancing the development of personalized cancer vaccines tailored to the unique antigenic profile of an individual’s tumor, offering a new frontier in cancer treatment [110].

The successful translation of pVLPs from research to clinical and industrial applications will require interdisciplinary collaborations among scientists, engineers, regulatory bodies, and industry stakeholders. Meeting stringent regulatory standards for safety and efficacy is essential for their adoption in clinical settings. To achieve this, future efforts should focus on establishing standardized protocols for pVLP production and characterization, and comprehensive preclinical and clinical studies to evaluate their real-world performance [12]. Such collaborations will also be crucial in addressing technical challenges and navigating the complex regulatory landscape that governs biotechnological innovations in medicine and agriculture [113].

## 4. Conclusions

In conclusion, exploring pVLPs presents a promising frontier in biotechnology and biomedical applications, offering significant advantages due to their structural versatility, safety, and potential for functionalization. Despite the extensive research on VLPs derived from mammalian viruses, pVLPs still need to be utilized, leaving substantial opportunities for innovation, particularly in therapeutic and diagnostic contexts. The critical influence of size, shape, and surface charge on the interaction of pVLPs with cells underscores the need for a more comprehensive understanding of these factors to optimize their design for specific applications. Furthermore, the scalability and production efficiency of pVLPs, especially when considering expression systems, remain key challenges that need to be addressed to harness their potential fully in industrial settings. Moving forward, interdisciplinary collaboration and a concerted effort to bridge the knowledge gap in pVLP research will be essential for developing advanced pVLP-based technologies. By integrating insights into the physicochemical properties, surface modifications, and biological interactions of pVLPs, future research can pave the way for groundbreaking advancements in medicine, agriculture, and beyond, ultimately contributing to global health and well-being.
